# Polymorphism of Baculoviral Inhibitor of Apoptosis Repeat-Containing 5 (BIRC5) Can Be Associated with Clinical Outcome of Non-Small Cell Lung Cancer

**DOI:** 10.3390/cells11060956

**Published:** 2022-03-10

**Authors:** Michał Szczyrek, Radosław Mlak, Aneta Szudy-Szczyrek, Kamila Wojas-Krawczyk, Karolina Kędziora, Janusz Milanowski

**Affiliations:** 1 Department of Pneumonology, Oncology and Allergology, Medical University of Lublin, 20-059 Lublin, Poland; kamila.wojas-krawczyk@umlub.pl (K.W.-K.); janusz.milanowski@umlub.pl (J.M.); 2 Chair and Department of Human Physiology, Medical University of Lublin, 20-080 Lublin, Poland; radoslaw.mlak@umlub.pl; 3 Chair and Department of Haematooncology and Bone Marrow Transplantation, Medical University of Lublin, 20-059 Lublin, Poland; aneta.szudy-szczyrek@umlub.pl; 4 Collegium Medicum, University of Zielona Góra, 65-417 Zielona Góra, Poland; karolina0kedziora@gmail.com

**Keywords:** BIRC5, Survivin, NSCLC, outcome

## Abstract

Non-small cell lung cancer (NSCLC) comprises about 85% of all lung cancers. Currently, NSCLC therapy is based on the analysis of specific predictors, whose presence qualifies patients for appropriate treatment. Baculoviral inhibitor of apoptosis repeat-containing 5 (BIRC5), also known as “survivin”, is a protein whose expression is characteristic for most malignant tumors and fetal tissue, while absent in mature cells. The biological role of BIRC5 is to counteract apoptosis by inhibiting the initiating and effector activities of caspases and binding to microtubules of the mitotic spindle. In our study, we looked for a relationship between *BIRC5* gene polymorphism and the effectiveness of platinum-based chemotherapy. The study group consisted of 104 patients with newly diagnosed locally advanced or metastatic NSCLC. DNA was isolated from pretreatment blood samples, and SNPs of *BIRC5* gene were analyzed. All patients received first-line platinum-based chemotherapy. Univariate analysis showed that a specific BIRC5 genotype was significantly associated with a higher risk of early progression (homozygous GG vs. heterozygous CG or CC: 28.9% vs. 11.9%). The presence of a homozygous GG genotype of the *BIRC5* gene was insignificantly related to PFS shortening and TTP shortening. Moreover, significantly higher risk of overall survival shortening was associated with the *BIRC5* homozygous GG genotype. Thus, studies on polymorphisms of selected genes affecting apoptosis may have a practical benefit for clinicians who monitor and treat NSCLC.

## 1. Introduction

Lung cancer is one of the biggest challenges of modern oncology. It is currently the leading cause of cancer deaths in the world. About 85% of all lung cancer cases are non-small cell lung cancer (NSCLC) [1]. Early diagnosis of NSCLC with surgical resection is associated with favorable prognosis, a 5-year survival rate of up to 90% for small, localized tumors defined as stage I. However, a majority of NSCLC patients (approximately 75%) are being diagnosed in the advanced stage (III/IV) of the disease and, despite significant improvement in oncology treatment, their outcomes remain poor. It is estimated that for advanced NSCLC patients, the 5-year survival rate is approximately 5% for patients with stage IIIB and only 1% for patients with stage IV [2]. There is an unmet medical need for the treatment of advanced NSCLC patients. Unfortunately, only 10–12% of Caucasian NSCLC tumor tissue expressed at least one predictive factor that predisposes those patients to receive molecularly targeted therapy or immunotherapy [3,4]. The understanding of the genetic mechanisms underlying cancer, as well as technological advances, have changed our perception of molecular disorders involved in NSCLC pathogenesis. It seems that the future research direction for NSCLC patients without predictive factors could be focused on chosen genetic factors or polymorphisms that would facilitate the decision over including these patients for chemotherapy treatment. More importantly, it would also assess the effectiveness of such treatment.

Baculoviral inhibitor of apoptosis repeat-containing 5 (BIRC5), also known as “survivin”, a protein encoded by the *BIRC5* gene, is a member of the inhibitor of apoptosis (IAP) family. The protein was discovered in 1997 by Ambrosini et al. in human B-cell lymphoma [5]. The biological role of BIRC5 is to counteract apoptosis by inhibiting the initiating and effector activities of caspases and binding to microtubules of the mitotic spindle. Survivin expression is characteristic for most malignant tumors and fetal tissue, while absent in mature cells. Disruption of surviving-related pathways leads to an increase of apoptosis and slows down tumor growth [6]. This implies survivin could be a potential therapeutic target, and a predictive factor for cancer therapies [7].

*BIRC5* gene is located on chromosome 17q25 with a length of 15 kb. Accumulating evidence suggests that single nucleotide polymorphisms (SNPs) of oncogenes or tumor suppressor genes could play a key role in carcinogenesis and tumor progression. *BIRC5* gene expression can be modified by a variety of factors, including SNPs. Many studies have assessed the relationship between *BIRC5* polymorphisms and the occurrence of neoplasms [8,9,10,11]. However, there are still limited data concerning the role of *BIRC5* polymorphisms on NSCLC therapy.

In this study, we assessed the presence of polymorphic variant rs8073069 of the *BIRC5* gene and its relationship with the clinical and demographic features, as well as with therapy effectiveness in advanced NSCLC patients without driver mutations.

## 2. Material and Methods

### 2.1. Clinical Characteristic of Study Population

We followed the methods described in previous publications (Szczyrek et al., 2021 [12]). The study group consisted of 104 Caucasian patients with newly diagnosed locally advanced or metastatic NSCLC. The staging of disease was determined according to the TNM classification (VII edition), and response to treatment was evaluated according to RECIST criteria (version 1.1). The first and second evaluations were performed after two and four cycles of chemotherapy. Performance status of patients was assessed with an ECOG-WHO scale. All patients received first-line chemotherapy with platinum-based regimens after earlier exclusion of the predictive factor presence (*EGFR* mutations, *ALK* or *ROS1* genes rearrangement, PD-L1 expression, according to the histopathological diagnosis). The detailed characteristics of patients are presented in Table 1.

### 2.2. Single Nucleotide Polymorphism Analysis of BIRC5 Gene

Prior to starting chemotherapy, 5 mL of whole blood were drawn from all participants and stored in −80 °C until further analyses were performed. DNA Blood Mini Kit (Qiagen, Canada) was used to isolate DNA. The quality and quantity of DNA were assessed using a NanoDrop Lite Spectrophotometer (Thermo Fisher Scientific, Waltham, MA, USA). The evaluation of SNPs of the *BIRC5* gene was performed using a real-time PCR method with allelic discrimination software. The Genotyping Master Mix and TaqMan probes (Applied Biosystems, Waltham, MA, USA) specific for studied SNPs (Thermo Fisher Scientific) were used for DNA amplification according to the manufacturer’s protocol in the RT7500 Real-time PCR device (Applied Biosystems, USA). All sample tests were run in triplicate.

### 2.3. Statistical Analysis

All analyses were performed using MedCalc 15.8 (MedCalc Software, Ostend, Belgium). Data were expressed as a percentage (for the categorized variable), median, and range (for continuous variables) because of non-normal data distribution (evaluated by D’Agostino-Pearson test). Risk of early progression was assessed with the use of an odds ratio test, whereas logistic regression was used for multivariate analysis. Univariate analysis of the risk of PFS, TTP, and OS shortening was performed with the use of the Kaplan-Meier estimation method (log-rank), whereas Cox logistic regression models were used in multivariate analysis with statistically significant factors from univariate analysis (α < 0.05) as included variables. In all analyses, we considered *p* values below 0.05 to be statistically significant.

The study was performed based on the approval of the institutional research committee (Bioethical Commission of Medical University of Lublin; consent reference number KE-0254/219/2015), in accordance with the 1964 Helsinki Declaration and its later amendments. All patients gave informed consent to participate in the study.

## 3. Results

### 3.1. Characteristics of Study Population

The study group was dominated by men (75%), patients aged 44 to 83 (median: 66) years, with an ECOG performance status of 1 (56.7%), at a higher stage of disease (68.3%), and with distant metastases (56.7%). The most common histological diagnoses were squamous (51.9%) and adenocarcinoma (41.4%). Treatment regimens were as follows: cisplatin with vinorelbine (60.6%), cisplatin with pemetrexed (24%), and cisplatin with gemcytabine (15.4%). Detailed demographic and clinical characteristic of the study group is presented in Table 1.

### 3.2. Distribution of Survivin Genotypes within the Study Group

A single nucleotide polymorphism defined as rs8073069 is located in the promoter sequence (−625 G > C) of the *BIRC5* gene and refers to the nucleotide transversion of guanine (wild variant) on cytosine (altered). None of demographic and clinical variables were significantly associated with the distribution of different *BIRC5* genotypes. (Supplementary material—Table S1. Distribution of survivin (rs8073069) genotypes according to demographic and clinical factors.).

### 3.3. Association of Survivin Polymorphism with the Risk of Early Progression in Study Population

What was adjusted by the statistically significant results of univariate analysis of clinical and genetic factors significantly associated with a higher risk of early progression (evaluation after 2 cycles of chemotherapy) included: higher disease stage (III vs. IVA: 25.8% vs. 7.9%, OR= 4.71, 95% CI: 1.24–17.92; *p* = 0.0231), presence of distant metastases (yes vs. no: 27.1% vs. 8.9%, OR = 3.50; 95%CI: 1.21–10.07; *p* = 0.0204), and specific *BIRC5* genotype (homozygous GG vs. heterozygous CG or CC: 28.9% vs. 11.9%, OR = 3.50, 95% CI: 1.21–10.07, *p* = 0.0204). On the other hand, the presence of the CG genotype was significantly related to lower risk of early progression (heterozygous CG vs. homozygous CC or GG: 10.4% vs. 26.8%; OR = 0.28, 9%%CI: 0.09–0.86; *p* = 0.0261). Detailed data on the influence of demographic, clinical, and genetic factors on the risk of early progression in the study group are presented in Table 2.

### 3.4. Association of Survivin Polymorphism with Survival Parameters in Study Populations

**Figure 1:**
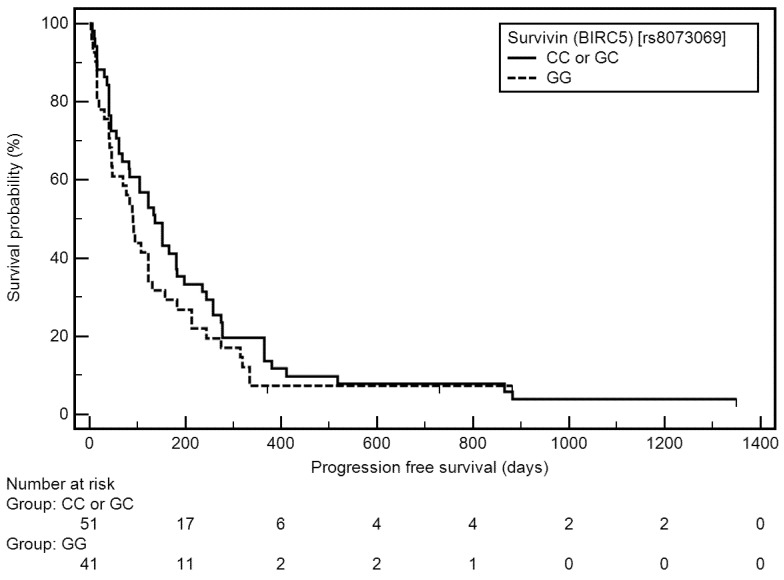
Kaplan–Meier curves representing probability of PFS shortening depending on *BIRC5* genotype.

Among the studied demographic, clinical, and genetic factors, the following were associated with a higher risk of progression-free survival (PFS) shortening: higher stage of disease (IV: median: 61 vs. 198 days; OR = 2.64, 95% CI: 1.73–4.02, *p* < 0.0010), higher number of pack-years (≥45: medians: 89 vs. 122 days; OR = 1.53, 95% CI: 1.00–2.34, *p* = 0.0374), cisplatin- and pemetrexed-based therapy as the first line chemotherapy (medians: 84 vs. 122 days; OR = 1.67, 95% CI: 0.96–2.91, *p* = 0.0263), lower number of cycles (1–3: medians: 41 vs. 213 days; OR = 2.31, 95% CI: 1.46–3.64, *p* < 0.0001), presence of distant metastases (yes: medians: 61 vs. 213 days; OR = 2.42, 95% CI: 1.51–3.88, *p* = 0.0005), including its presence in CNS (yes: medians: 41 vs. 198 days; OR = 4.69, 95% CI: 1.43–15.36, *p* < 0.0001), in bones (yes: medians: 47 vs. 198 days; OR = 2.34, 95% CI: 1.20–4.56, *p* = 0.0014), in second lung (yes: medians: 69 vs. 198 days; OR = 2.19, 95% CI: 1.08–4.43, *p* = 0.0057), in adrenal glands (yes: medians: 40 vs. 198 days; OR = 6.73, 95% CI: 1.11–40.75, *p* < 0.0001), in liver (yes: medians: 61 vs. 198 days; OR = 5.49, 95% CI: 1.07–28.25, *p* < 0.0001), and in distant lymph nodes (yes: medians: 69 vs. 198 days; OR = 2.11, 95% CI: 0.98–4.54, *p* = 0.0134), and longer time from diagnosis to start of treatment (≥17 days: medians: 92 vs. 122 days; OR = 1.58, 95% CI: 1.03–2.42, *p* = 0.0254). In context of genetic examination, the homozygous GG genotype of the *BIRC5* gene was insignificantly related to PFS shortening (Figure 1).

**Figure 2:**
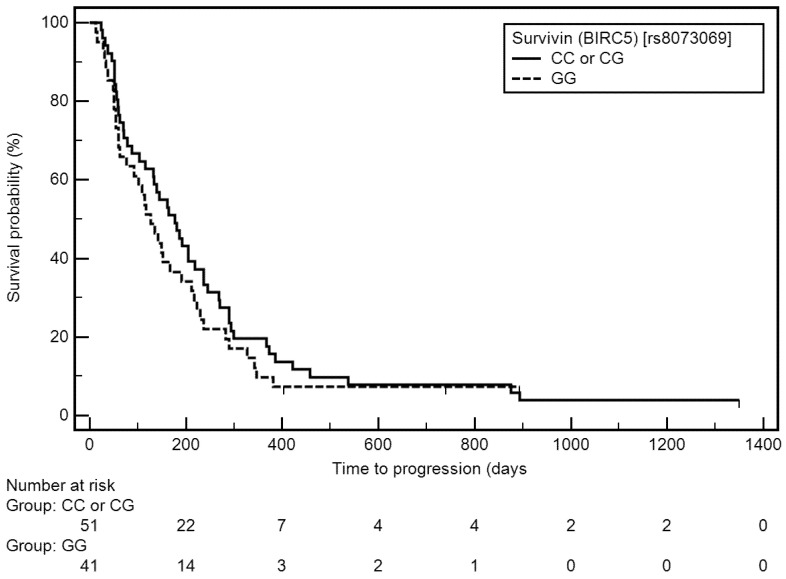
Kaplan–Meier curves representing probability of TTP shortening depending on *BIRC5* genotype.

However, a higher risk of time to progression (TTP) shortening was associated with a higher stage of disease (IV: medians: 92 vs. 222 days; HR = 2.60, 95% CI: 1.71–3.96, *p* < 0.0010), higher number of pack-years (≥45: medians: 115 vs. 177 days; HR = 1.57, 95% CI: 1.02–2.40, *p* = 0.0292), use of cisplatin- and pemetrexed-based therapy as first line chemotherapy (medians: 92 vs. 151 days; HR = 1.74, 95% CI: 0.99–3.03, *p* = 0.0185), lower number of cycles (1–3: medians: 60 vs. 237 days; HR = 2.15, 95% CI: 1.37–3.38, *p* = 0.0002), presence of distant metastases (medians: 92 vs. 237 days; HR = 2.48, 95% CI: 1.55–3.97, *p* = 0.0004), including its presence in CNS (yes: medians: 60 vs. 222 days; HR = 3.54, 95% CI: 1.23–10.16, *p* = 0.0001), in bones (yes: medians: 70 vs. 222 days; HR = 2.25, 95% CI: 1.17–4.35, *p* = 0.0025), in second lung (yes: medians: 92 vs. 222 days; HR = 2.15, 95% CI: 1.07–4.34, *p* = 0.0075), in adrenal glands (yes: medians: 58 vs. 222 days; HR = 7.38, 95% CI: 1.13–48.39, *p* < 0.0001), in liver (yes: medians: 70 vs. 222 days; HR = 6.33, 95% CI: 1.10–36.40, *p* < 0.0001), and in distant lymph nodes (yes: medians: 127 vs. 222 days; HR = 2.04, 95% CI: 0.96–4.36, *p* = 0.0194). The GG genotype of the *BIRC5* gene was insignificantly related to TTP shortening (Figure 2).

**Figure 3:**
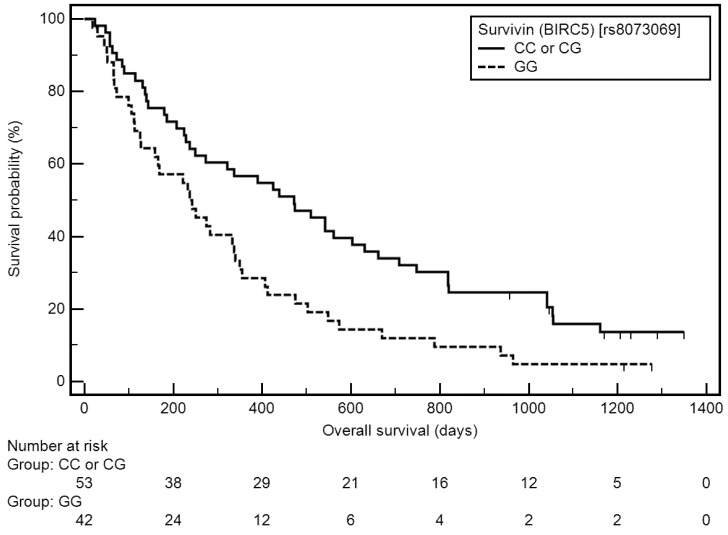
Kaplan–Meier curves representing probability of OS shortening depending on *BIRC5* genotype.

Similarly, a higher risk of overall survival (OS) shortening was associated with a higher stage of disease (IV: medians: 233 vs. 502 days; HR = 1.99, 95% CI: 1.30–3.04, *p* = 0.0018), lower number of cycles (1–3: medians: 143 vs. 502 days; HR = 1.74, 95% CI: 1.11–2.71, *p* = 0.0094), presence of distant metastases (medians: 224 vs. 542 days; HR = 2.25, 95% CI: 1.39–3.62, *p* = 0.0019) including its in CNS (yes: medians: 60 vs. 222 days; HR = 4.24, 95% CI: 1.28–14.05, *p* < 0.0001), in bones (yes: medians: 70 vs. 222 days; HR = 2.19, 95% CI: 1.22–4.28, *p* = 0.0041), in second lung (yes: medians: 92 vs. 222 days; HR = 1.89, 95% CI: 0.95–3.79, *p* = 0.0304), in adrenal glands (yes: medians: 58 vs. 222 days; HR = 2.51, 95% CI: 0.79–7.96, *p* = 0.0194), and in distant lymph nodes (yes: medians: 127 vs. 222 days; HR = 1.99, 95% CI: 0.91–4.33, *p* = 0.0290), and the *BIRC5* homozygous GG genotype (medians: 236 vs. 472 days; HR = 1.77, 95% CI: 1.13–2.78, *p* = 0.0066; Figure 3). PFS, TTP, and OS according to demographic, clinical, and genetic factors are presented in Table 3.

### 3.5. Multivariate Analysis of Survivin Polymorphism Associated with Survival Parameters in Study Population

Multivariate analysis revealed that independent factors associated with a lower risk of PFS shortening include younger age (<66.5 years: HR = 0.45, 95% CI: 0.26–0.77, *p* = 0.0040), higher number of CTH cycles (>3: HR = 0.33, 95% CI: 0.20–0.56, *p* < 0.0001), distant metastases in CNS (yes: HR = 6.45, 95% CI: 2.86–14.57, *p* < 0.0001), in bones (yes: HR = 2.91, 95% CI: 1.59–5.31, *p* = 0.0005), in second lung (yes: HR = 2.97, 95% CI: 1.40–6.29, *p* = 0.0046), in adrenal glands (yes: HR = 12.15, 95% CI: 3.95–37.38, *p* < 0.0001), in liver (yes: HR = 8.03, 95% CI: 2.58–24.99, *p* = 0.0003), and in distant lymph nodes (yes: HR = 3.13, 95% CI: 1.46–6.71, *p* = 0.0035), and the CC genotype of the *BIRC5* gene (HR = 0.28, 95% CI: 0.10–0.80, *p* = 0.0181). In contrast, higher risk of PFS shortening was independently associated with the histopathological diagnosis of squamous cell carcinoma (HR = 2.85, 95% CI: 1.24–6.54, *p* = 0.0138) and the GG genotype of the *BIRC5* gene (HR = 1.92, 95% CI: 1.15–3.24, *p* = 0.0136).

Independent factors associated with a lower risk of TTP shortening were younger age (<66.5 years: HR = 0.49, 95% CI: 0.29–0.84, *p* = 0.0092), higher number of chemotherapy cycles (>3: HR = 0.36, 95% CI: 0.22–0.60, *p* = 0.001), and the CC genotype of the *BIRC5* gene (HR = 0.28, 95% CI: 0.10–0.76, *p* = 0.0125). However, the presence of distant metastases (HR = 3.76, 95% CI: 1.09–12.89, *p* = 0.0363), including its presence in CNS (yes: HR = 4.22, 95% CI: 1.93–9.21, *p* = 0.0003), in bones (yes: HR = 2.92, 95% CI: 1.59–5.37, *p* = 0.0006), in second lung (yes: HR = 3.16, 95% CI: 1.15–6.81, *p* = 0.0035), in adrenal glands (yes: HR = 17.14, 95% CI: 4.95–59.37, *p* < 0.0001), in liver (yes: HR = 10.88, 95% CI: 3.33–35.56, *p* = 0.0001), and in distant lymph nodes (yes: HR = 3.09, 95% CI: 1.44–6.63, *p* = 0.0040), histopathological diagnosis of squamous cell carcinoma (HR = 3.09, 95% CI: 1.35–7.07, *p* = 0.0077), and the GG genotype of the *BIRC5* gene (HR = 1.74, 95% CI: 1.05–2.89, *p* = 0.0332) were significantly related to higher risk of TTP shortening.

The only independent prognostic factor associated with reduction of risk of OS shortening was a higher number of chemotherapy cycles (>3: HR = 0.49, 95% CI: 0.30–0.81, *p* = 0.0056), distant metastases in CNS (yes: HR = 6.18, 95% CI: 2.64–14.45, *p* < 0.0001), in bones (yes: HR = 2.33, 95% CI: 1.31–4.13, *p* = 0.0042), in second lung (yes: HR = 3.36, 95% CI: 1.61–7.00, *p* = 0.0013), in adrenal glands (yes: HR = 3.40, 95% CI: 1.37–8.43, *p* = 0.0085), and in distant lymph nodes (yes: HR = 3.19, 95% CI: 1.45–7.02, *p* = 0.0040). The factors that independently increased the risk of OS shortening included diagnosis of squamous cell carcinoma (HR = 2.10, 95% CI: 1.04–4.28, *p* = 0.0406) and GG genotype of the *BIRC5* gene (HR = 3.63, 95% CI: 2.05–6.43, *p* < 0.0001). Results of Cox’s logistic regression analysis for PFS, TTP, and OS are presented in Table 4.

## 4. Discussion

Survivin is of great interest as a biomarker for the assessment of prognosis in neoplastic disease. However, the observations regarding its prognostic value are controversial. So far, a relationship has been demonstrated between high protein expression and unfavorable prognosis in cancers of the esophagus, liver, ovary, bile ducts, and endometrium. On the other hand, it was observed that in the cases of gastric, bladder, and breast cancer, ependymoma, pancreatic adenocarcinoma, and osteosarcoma, a favorable prognosis is associated with high expression [13,14].

Studied SNP is located in the regulatory region (promoter) of *BIRC5* and is predicted to change the transcription factor binding and microRNA binding site by the Pupasuite prediction tool. The promoter SNP rs8073069 is located at position –625 from the translation start site and several MYB binding sites are reported in the neighboring regions [15]. Genetic variation upstream of the BIRC5 gene has also been reported to strongly correlate with BIRC5 expression by changing the binding motif of the CDE/CHR repressor element or by creating an alternative binding site for the transcription factor GATA-1 [16].

The importance of *BIRC5* in predicting the course of NSCLC has been a subject of many studies, which were summarized in two meta-analyses. Zhang et al., in a meta-analysis of 2703 NSCLC patients from 28 studies, showed that survivin overexpression is associated with a shorter survival (HR = 2.03, 95% CI: 1.78–2.33, Egger’s test, *p* = 0.24). No significant heterogeneity was observed between the analyzed studies (I2 = 26.9%). Survivin was identified as a prognostic marker in advanced NSCLC (HR = 1.93, 95% CI: 1.49–2.51), but not in early stages of the disease (HR = 1.97, 95% CI: 0.76–5.14). The high level of protein expression in patients with stage III and IV NSCLC correlated with a poor prognosis [17].

In a later meta-analysis conducted based on the results obtained from 29 published studies of 2517 patients with NSCLC, Huang et al. reached similar conclusions. The analysis showed that overexpression of survivin in NSCLC patients is a negative prognostic factor for survival (HR 1.95, 95% CI: 1.65–2.29; *p* < 0.001). There was no heterogeneity in the stratification by histology types. After adjusting for publication error, the results remained significant (HR = 1.71, 95% CI: 1.44–2.02, *p* < 0.001) [18].

Survivin’s mechanism of action in the pathogenesis of NSCLC has been suggested to be through the regulation of cell proliferation, cell cycle, apoptosis, and colony formation. Zhang et al. assessed the effects of in vivo and in vitro expression of the protein on cells of the human A549 NSCLC line. Reduction of surviving expression in A549 tumor cells using the RNA quenching method significantly suppressed the proliferation and colony formation capacity of cells and induced tumor apoptosis in vitro. Mice inoculated with A549 cells developed cancer. Interestingly, treatment with shRNA (small hairpin RNA) targeting survivin significantly inhibited tumor growth, with no apparent adverse effects. The authors thus suggested that suppression of survivin expression by RNA interference may induce NSCLC apoptosis, which in turn may allow a novel approach to anti-cancer gene therapy [19].

Another research goal was to look for factors regulating the level of survivin expression. Particular attention was paid to polymorphisms of the promoter region of the protein-encoding gene. Several SNPs that specifically marked this region have been identified so far [20]. The rs8073069 locus (−625 G/C) is one of the polymorphic sites in the promoter region of the *BIRC5* gene. The selected SNP has a proven effect in promoting susceptibility to cancer, as well as affecting its course.

Yang et al. proved that the rs8073069-C allele is a risk factor for esophageal squamous cell carcinoma (ESCC), the OR of the CC genotype compared to the GG genotype was 2.404. The authors observed differences in the levels of survivin expression in subgroups of patients with different rs8073069 G/C variants. They suggested that the rs8073069 G/C polymorphism was associated with susceptibility to ESCC, possibly by influencing survivin expression [21].

Li et al. investigated the relationship between the rs8073069 SNP and the risk of hepatocellular carcinoma (HCC) in the Han Chinese population. They recruited a group of 178 HCC patients and 196 healthy volunteers. They found no relationship between the studied polymorphism and risk of HCC, but noted that rs8073069G polymorphism was probably one of the protective haplotypes for HCC [22].

Currently, only limited data are available regarding the role of rs8073069 SNP of the *BIRC5* gene in NSCLC pathogenesis. In a study by Jang et al., eight selected SNPs were genotyped in a group of 582 healthy volunteers and 582 lung cancer patients from the Korean population. There was no relationship between the rs8073069 G/C polymorphism and the risk of lung cancer. Instead, they observed that the −625G/−31G/9194A/9809T haplotype carrying the −31G allele was associated with a significant reduction in the risk of adenocarcinoma (adjusted OR = 0.56, 95% CI = 0.40–0.77, *p* = 0.0004) [23].

The overexpression of BIRC5 in human malignancies is defined as one of the most important factors for the progression and chemoresistance of tumors. In various studies, it was correlated with a more aggressive course of the disease and poor prognosis, which suggests that survivin could have prognostic, as well as therapeutic, implications. A study by Dai et al. had shown the association between polymorphisms of the *BIRC5* gene and the prognosis in NSCLC. They genotyped 12 SNPs of the survivin gene in 568 NSCLC patients and demonstrated that the rs8073069 polymorphism had an impact on the survival of those patients. The homozygous GG genotype was associated with a worse prognosis compared to CG/CC (HR = 1.76, 95% CI: 1.16–2.67). In a subgroup of 185 patients in stage III or IV treated exclusively with chemotherapy, SNP rs8073069 also had prognostic significance. A significantly shorter median survival time was observed in homozygous GG genotype carriers versus CG/CC carriers (HR = 2.06, 95% CI: 1.10–3.87) [24]. BIRC5 is expressed in the majority of human cancers, including those of squamous histology, e.g., head and neck, laryngeal, esophageal, lung, ovarian, gastric, colorectal, bladder, pancreatic, and prostate cancer, as well as melanoma and soft tissue sarcomas. Among other apoptotic-related proteins (p53, Bcl-2, Bax, COX-2), it has a potential impact on the functioning of the cell cycle. A study by Porębska et al. [25] had evaluated the relationship between apoptosis markers, examined by immunohistochemical method, and clinicopathological parameters in lung adenocarcinoma (LUAD) and squamous cell carcinoma (LUSC). They found significantly more frequent expression of Bax and BIRC5 in LUAD than LUSC. However, there was no correlation between the apoptosis markers and gender, the presence of vessel emboli or TNM characteristic, except for Bcl-2. A greater variability in markers expression was seen in LUAD than LUSC. However, we should keep in mind that this study used an assessment of the surface expression of the investigated markers at the protein level. A large sample size is needed to draw the conclusions regarding the differences in the expression of apoptosis markers between LUAD and LUSC and their potential role.

In our study, the results are in keeping with those described in the literature. The presence of a homozygous GG genotype was associated with a higher risk of progression after two cycles of chemotherapy (GG vs. CG or CC: 28.9% vs. 11.9%, *p* = 0.0204) and with a higher risk of OS shortening (medians: 236 vs. 472 days; *p* = 0.0066). In a multivariate analysis, we showed that the GG genotype was independently associated with a higher risk of PFS shortening (OR = 1.92, 95% CI: 1.15–3.24, *p* = 0.0136), as well as OS shortening (OR = 3.63, 95% CI: 2.05–6.43, *p* < 0.0001).

It is suggested that the localization of the rs8073069 SNP in the promoter region may inhibit survivin transcription. Thus, survivin acts as an inhibitor of apoptosis, which is necessary for the elimination of mutated or transformed cells from the body, and it seems likely that individuals with a higher survivin expression may have a reduced ability to eliminate DNA-damaged cells and, therefore, have shorter survival and worse prognosis in the case of cancer [24,26].

## 5. Conclusions

Studies on polymorphisms of selected genes may have a practical benefit for clinicians who monitor and treat NSCLC, especially in patients with particular SNPs that predispose to rapid progression. According to our study, the rs8073069 SNP of the *BIRC5* gene can be a useful marker in predicting treatment outcome in NSCLC patients. The methodology of such testing is relatively simple and easy to interpret, which may facilitate its introduction into the clinic. In the future, it would be advisable to undertake research to identify the detailed mechanisms underlying these results.

## Figures and Tables

**Table 1:** Characteristics of the study group.

Variable		Study Group
		*n* = 104
Sex	Male	78 (75%)
	Female	26 (25%)
Age [years], median (range)		66 (44–83)
	≥66.5	51 (49%)
	<66.5	53 (51%)
Disease stage	III	38 (31.7%)
	IV	66 (68.3%)
Distant metastases	No	45 (43.3%)
	Yes	59 (56.7%)
Histopathology	AC	43 (41.4%)
	SCC	54 (51.9%)
	NOS	7 (6.7%)
Performance status (ECOG score)	0	18 (17.3%)
	1	59 (56.7%)
	2	27 (26%)
Smoking status	Smoker	97 (93.3%)
	Non-smoker	7 (6.7%)
Packyears [years], median (range)		45 (1–100)
	≥45	47 (45.1%)
	<45	57 (54.8%)
Weight [kg], median (range)		73.5 (46–117)
BMI [kg/m^2^], median (range)		24.82 (15.02–156.55)
	≥25	56 (53.9%)
	<25	48 (46.1%)
Body weight loss (%), median (range)		7 (0–25)
	≥10%	35 (33.7%)
	<10%	69 (66.3%)
Time from diagnosis to treatment [days]		16 (3–217)
	≥17	49 (47.1%)
	<17	55 (52.9%)
First line chemotherapy	Cp-PEM Cp-V Cp-G	25 (24%) 63 (60.6%) 16 (15.4%)
Number of cycles	1–3 4–8	53 (51%) 51 (49%)
First evaluation after first line chemotherapy	PR SD PD	43 (41.4%) 41 (39.4%) 20 (19.2%)
Second evaluation after first line chemotherapy	PR SD PD	10 (19.6%) 33 (64.7%) 8 (15.7%)
Occupational exposure	No Yes	90 (86.5%) 14 (13.5%)
Family history of any cancer	No Yes	48 (46.1%) 56 (53.9%)
Family history of lung cancer	No Yes	77 (74%) 27 (26%)

Abbreviations: AC—adenocarcinoma; BMI—body mass index; Cp—Cisplatin, G—Gemcitabine; NOS—not otherwise specified; PD—progressive disease; PEM—Pemetrexed; PR—partial response; SCC—squamous cell carcinoma; SD—stable disease; V—Vinorelbine.

**Table 2:** Risk of early progression according to demographic, clinical, and genetic factors.

Variable		PD	PR and SD	*Univariate*	*Multivariate ^#^*
		*n* = 20 (19.2%)	*n* = 84 (80.8%)	OR [95% CI] *p*	OR [95% CI] *p*
Sex	Male	15 (19.2%)	63 (80.8%)	1.00 [0.32–3.08] 1.0000	0.98 [0.29–3.32] 0.9733
	Female	5 (19.2%)	21 (80.8%)		
Age [years]	≥66.5	12 (23.5%)	39 (76.5%)	1.73 [0.64–4.66] 0.2785	1.86 [0.64–5.43] 0.2565
	<66.5	8 (15.1%)	45 (84.9%)		
Disease stage	IV	17 (25.8%)	49 (74.2%)	4.05 [1.10–14.88] 0.0353 *	4.71 [1.24–17.92] 0.0231 *
	III	3 (7.9%)	35 (92.1%)		
Distant metastases	Yes	16 (27.1%)	43 (72.9%)	3.81 [1.18–12.36] 0.0257 *	3.50 [1.21–10.07] 0.0204 *
	No	4 (8.9%)	41 (91.1%)		
Performance status (ECOG score)	1 or 2	17 (19.8%)	69 (80.2%)	1.23 [0.32–4.74] 0.7618	0.48 [0.10–2.24] 0.3494
	0	3 (16.7%)	15 (83.3%)		
Histopathology (I)	AC	11 (25.6%)	32 (74.4%)	1.99 [0.74–5.31] 0.1721	1.62 [0.56–4.65] 0.3734
	SCC or NOS	9 (14.8%)	52 (85.2%)		
	SCC	9 (16.7%)	45 (83.3%)	0.71 [0.27–1.89] 0.4916	0.77 [0.26–2.23] 0.6272
	AC or NOS	11 (22%)	39 (78%)		
Smoking status	Smoker	17 (17.5%)	80 (82.5%)	0.29 [0.06–1.38] 0.1191	0.31 [0.06–1.66] 0.1709
	Non-smoker	3 (42.9%)	4 (57.1%)		
Packyears [years]	≥45	7 (14.9%)	40 (85.1%)	0.58 [0.21–1.57] 0.2910	
	<45	13 (23.2%)	44 (76.8%)		
BMI [kg/m^2^]	≥25	9 (19.1%)	39 (80.9%)	0.94 [0.35–2.51] 0.9083	
	<25	11 (19.6%)	45 (80.4%)		
Body weight loss [%]	≥10%	11 (15.9%)	58 (84.1%)	0.55 [0.20–1.48] 0.2359	1.64 [0.56–4.82] 0.3702
	<10%	9 (25.7%)	26 (74.3%)		
Time from diagnosis to treatment [days]	≥17	10 (20.4%)	39 (79.6%)	1.15 [0.43–3.06] 0.7738	1.09 [0.39–3.08] 0.8644
	<17	10 (18.2%)	45 (81.8%)		
Occupational exposure	Yes	2 (14.3%)	12 (85.7%)	0.67 [0.14–3.25] 0.6158	0.51 [0.09–2.69] 0.4248
	No	18 (%)	72 (%)		
Family history of any cancer	Yes	13 (23.2%)	43 (76.8%)	1.77 [0.64–4.88] 0.2692	1.87 [0.64–5.50] 0.2525
	No	7 (14.6%)	41 (85.4%)		
Family history of lung cancer	Yes	7 (25.9%)	20 (74.1%)	1.72 [0.60–4.91] 0.3084	2.05 [0.66–6.39] 0.2165
	No	13 (16.9%)	64 (83.1%)		
First line chemotherapy scheme	Cp-PEM	6 (24%)	19 (76%)	1.47 [0.50–4.34] 0.4892	1.16 [0.36–3.69] 0.8067
	Cp-V or Cp-G	14 (17.7%)	65 (82.3%)		
	Cp-V	10 (15.9%)	53 (84.1%)	0.58 [0.22–1.56] 0.2845	0.73 [0.25–2.13] 0.5641
	Cp-PEM or Cp-G	10 (24.4%)	31 (75.6%)		
	Cp-G	4 (25%)	12 (75%)	1.50 [0.43–5.26] 0.5264	1.37 [0.36–5.28] 0.6436
	Cp-PEM or Cp-V	16 (18.2%)	72 (81.8%)		
*BIRC5* (rs8073069) genotype	CC	2 (18.2%)	9 (81.8%)	0.92 [0.18–4.66] 0.9256	0.92 [0.18–14.81] 0.9216
	GG or CG	18 (19.3%)	75 (80.7%)		
	GG	13 (28.9%)	32 (71.1%)	3.02 [1.09–8.36] 0.0336 *	3.50 [1.21–10.07] 0.0204 *
	CC or CG	7 (11.9%)	52 (88.1%)		
	CG	5 (10.4%)	43 (89.6%)	0.32 [0.11–0.95] 0.0409 *	0.28 [0.09–0.86] 0.0261 *
	CC or GG	15 (26.8%)	41 (73.2%)		

Abbreviations: AC—adenocarcinoma; BMI—body mass index; CI—confidence interval; CP—Cisplatin, G—Gemcitabine; NOS—not otherwise specified; OR—odds ratio; PD—progressive disease; PEM—Pemetrexed; PR—partial response; SCC—squamous cell carcinoma; SD—stable disease; V—Vinorelbine. *—statistically significant result. ^#^—results of multivariate analysis were adjusted for disease stage (but not distant metastases as M characteristic is included in TNM stage) and *BIRC5* SNP.

**Table 3:** Progression-free survival, time to progression, and overall survival according to demographic, clinical, and genetic factors.

Variable		Progression-Free Survival		Time to Progression		Overall Survival	
		Median (days)	*p* HR (95% CI)	Median (days)	*p* HR (95%CI)	Median (days)	*p* HR (95% CI)
Sex	Male Female	41 122	0.7841 0.93 (0.56–1.55) 1.07(0.64–1.78)	164 60	0.7789 0.93(0.56–1.55) 1.07(0.64–1.78)	321 407	0.5208 1.18 (0.72–1.94) 0.84 (0.51–1.39)
Age [years]	≥66.5 <66.5	82 122	0.1053 1.40 (0.91–2.16) 0.71 (0.46–1.09)	117 177	0.1059 1.41 (0.92–2.16) 0.71 (0.46–1.09)	236 407	0.0577 1.50 (0.97–2.31) 0.67 (0.43–1.03)
Disease stage	III IV	198 61	<0.0010 * 0.38 (0.25–0.58) 2.64 (1.73–4.02)	222 92	<0.0010 * 0.38 (0.25–0.59) 2.60 (1.71–3.96)	502 233	0.0018 * 0.50 (0.33–0.77) 1.99 (1.30–3.04)
Performance status (ECOG score)	0 1 and 2	182 105	0.7049 0.86 (0.42–1.79) 1.16 (0.56–2.40)	190 139	0.8071 0.91 (0.43–1.91) 1.10 (0.52–2.31)	438 282	0.2111 0.61 (0.32–1.17) 1.63 (0.86–3.09)
Smoking status	Smoker Non-smoker	122 36	0.6115 0.79 (0.29–2.16) 1.26 (0.46–3.41)	149 51	0.6865 0.83 (0.31–2.22) 1.20 (0.45–3.20)	332 412	0.3914 1.48 (0.69–3.17) 0.68 (0.32–1.45)
Pack years	<45 ≥45	122 89	0.0374 * 0.65 (0.43–0.99) 1.53 (1.00–2.34)	177 115	0.0292 * 0.64 (0.52–0.97) 1.57 (1.02–2.40)	337 321	0.2597 0.78 (0.51–1.20) 1.27 (0.83–1.95)
1st line chemotherapy(I)	Cisplatin + Pemetrexed Other	841 22	0.0263 * 1.67 (0.96–2.91) 0.60 (0.34–1.04)	92 151	0.0185 * 1.74 (0.99–3.03) 0.57 (0.33–1.01)	233 337	0.0716 1.55 (0.89–2.67) 0.65 (0.37–1.20)
	PN Other	133 83	0.0770 0.69 (0.44–1.08) 1.45 (0.92–2.28)	167 117	0.1250 0.72 (0.46–1.13) 1.39 (0.88–2.18)	332 321	0.9832 0.99 (0.64–1.54) 1.00 (0.65–1.55)
	PG Other	76 122	0.9582 1.02 (0.54–1.93) 0.98 (0.52–1.86)	135 164	0.6860 0.88 (0.48–1.61) 1.14 (0.62–2.09)	355 337	0.5481 0.82 (0.44–1.52) 1.22 (0.66–2.27)
Number of cycles	≤3 >3	41 213	<0.0001 * 2.31 (1.46–3.64) 0.43 (0.27–0.68)	60 237	0.0002 * 2.15 (1.37–3.38) 0.46 (0.29–0.73)	143 502	0.0094 * 1.74 (1.11–2.71) 0.58 (0.37–0.90)
Distant metastases (any)	No Yes	213 61	0.0005 * 0.41 (0.26–0.66) 2.42 (1.51–3.88)	237 92	0.0004 * 0.40 (0.25–0.65) 2.48 (1.55–3.97)	542 224	0.0019 * 0.44 (0.27–0.71) 2.25 (1.39–3.62)
Distant metastases (CNS)	No Yes	198 41	<0.0001 * 0.21 (0.06–0.70) 4.69 (1.43–15.36)	222 60	0.0001 * 0.28 (0.09–0.81) 3.54 (1.23–10.16)	542 99	<0.0001 * 0.24 (0.07–0.78) 4.24 (1.28–14.05)
Distant metastases (Bones)	No Yes	198 47	0.0014 * 0.43 (0.22–0.83) 2.34 (1.20–4.56)	222 70	0.0025 * 0.44 (0.23–0.86) 2.25 (1.17–4.35)	542 185	0.0041 * 0.46 (0.23–0.89) 2.19 (1.22–4.28)
Distant metastases (second lung)	No Yes	198 69	0.0057 * 0.46 (0.23–0.93) 2.19 (1.08–4.43)	222 92	0.0075 * 0.46 (0.23–0.94) 2.15 (1.07–4.34)	542 159	0.0304 * 0.53 (0.26–1.06) 1.89 (0.95–3.79)
Distant metastases (andrenal glands)	No Yes	198 40	<0.0001 * 0.15 (0.02–0.90) 6.73 (1.11–40.75)	222 58	<0.0001 * 0.13 (0.02–0.89) 7.38 (1.13–48.39)	542 114	0.0194 * 0.40 (0.13–1.27) 2.51 (0.79–7.96)
Distant metastases (liver)	No Yes	198 61	<0.0001 * 0.18 (0.03–0.94) 5.49 (1.07–28.25)	222 70	<0.0001 * 0.16 (0.03–0.91) 6.33 (1.10–36.40)	542 137	0.2408 1.73 (0.54–5.58) 0.57 (0.18–1.86)
Distant metastases (distant lymph nodes)	No Yes	198 69	0.0134 * 0.47 (0.22–1.02) 2.11 (0.98–4.54)	222 127	0.0194 * 0.49 (0.23–1.04) 2.04 (0.96–4.36)	542 249	0.0290 * 0.50 (0.23–1.09) 1.99 (0.91–4.33)
Weight [kg]	≥74 kg <74 kg	122 92	0.9349 0.98 (0.65–1.50) 1.02 (0.67–1.55)	177 142	0.9347 0.98 (0.65–1.50) 1.02 (0.67–1.55)	339 274	0.4725 0.85 (0.56–1.31) 1.17 (0.76–1.79)
BMI [kg/m^2^]	≥24.91 <24.91	122 89	0.7448 1.07 (0.70–1.63) 0.93 (0.61–1.42)	142 151	0.6772 1.09 (0.72–1.66) 0.92 (0.60–1.39)	339 274	0.8440 0.96 (0.63–1.47) 1.04 (0.68–1.60)
Wieght loss [%]	<9.84 ≥9.84	131 47	0.4782 0.85 (0.53–1.36) 1.17 (0.73–1.87)	181 78	0.5073 0.86 (0.54–1.37) 1.16 (0.73–1.86)	438 168	0.0833 0.67 (0.41–1.11) 1.48 (0.90–2.45)
Time from diagnosis to treatment [days]	≥17 <17	92 122	0.0254 * 1.58 (1.03–2.42) 0.64 (0.41–0.97)	149 145	0.1736 1.33 (0.87–2.02) 0.75 (0.49–1.15)	339 282	0.8289 0.95 (0.62–1.46) 1.05 (0.68–1.60)
Occupational exposure	No Yes	95 105	0.6952 1.13 (0.62–2.08) 0.88 (0.48–1.62)	139 164	0.7607 1.10 (0.60–2.03) 0.91 (0.49–1.67)	282 350	0.9557 1.02 (0.54–1.91) 0.98 (0.52–1.84)
Family history of any cancer	No Yes	152.00 95.00	0.8875 0.97 (0.64–1.48) 1.03 (0.68–1.57)	190.00 134.00	0.8701 0.96 (0.63–1.47) 1.03 (0.68–1.58)	282.00 332.00	0.5718 1.13 (0.74–1.73) 0.88 (0.58–1.36)
Family history of lung cancer	No Yes	107.00 92.00	0.9243 1.03 (0.55–1.92) 0.97 (0.52–1.81)	135.00 132.00	0.8842 1.05 (0.56–1.95) 0.95 (0.51–1.78)	438.00 168.00	0.5715 0.84 (0.45–1.56) 1.19 (0.64–2.22)
Histopathology	SCC AC and NOS	95.00 137.00	0.9491 0.98 (0.58–1.66) 1.02 (0.60–1.72)	139.00 167.00	0.8259 0.94 (0.58–1.60) 1.06 (0.63–1.79)	389.00 337.00	0.8306 0.94 (0.54–1.64) 1.06 (0.61–1.85)
	AC SCC and NOS	137.00 89.00	0.6508 0.89 (0.53–1.49) 1.13 (0.67–1.89)	167.00 135.00	0.7572 0.92 (0.55–1.55) 1.08 (0.64–1.83)	337.00 389.00	0.7346 1.10 (0.63–1.92) 0.91 (0.52–1.59)
*BIRC5* (rs8073069)	CC GG and CG	61.00 122.00	0.1027 0.55 (0.30–1.01) 1.83 (0.99–3.36)	219.00 145.00	0.0871 0.52 (0.29–0.96) 1.90(1.04–3.47)	438.00 321.00	0.2093 0.59 (0.30–1.17) 1.68 (0.86–3.28)
	GG CC and CG	91.00 137.00	0.2788 1.26 (0.81–1.94) 0.79 (0.52–1.23)	127.00 177.00	0.3392 1.23 (0.80–1.89) 0.81 (0.53–1.25)	236.00 472.00	0.0066 * 1.77 (1.13–2.78) 0.56 (0.36–0.89)
	CG CC and CG	137.00 89.00	0.9644 1.01(0.66–1.54) 0.99 (0.65–1.51)	164.00 127.00	0.8070 1.05 (0.69–1.60) 0.94 (0.62–1.45)	473.00 242.00	0.0788 0.68 (0.45–1.05) 1.46 (0.95–2.24)

Abbreviations: AC—adenocarcinoma; BMI—body mass index; Cis—Cisplatin; Pem—Pemetrexed; PG—Cisplatin + Gemcitabine; PN—Cisplatin + Vinorelbine; SCC—squamous cell carcinoma. *-statistically significant. In some cases (*n* = 12 for PFS and TTP; *n* = 9 for OS) reliable determination of the survival time was not possible (too short follow-up—discontinuation of treatment prior to assessment time because of poor tolerance, failure to appear at the next appointment, or any and all contact with a patient was lost).

**Table 4:** Cox’s logistic regression analysis for progression-free survival, time to progression, and overall survival.

Variable		Progression-Free Survival	Time to Progression	Overall Survival
		*p* HR (95% CI)	*p* HR (95% CI)	*p* HR (95% CI)
Sex	Female	0.4043 1.29 (0.71–2.33)	0.3292 1.38 (0.75–2.39)	0.2946 1.36 (0.77–2.41)
Age [years]	<66.5	0.0040 * 0.45 (0.26–0.77)	0.0092 * 0.49 (0.29–0.84)	0.2146 0.72 (0.43–1.20)
Disease stage	IV	0.6042 0.72 (0.22–2.43)	0.4531 0.63 (0.19–2.09)	0.4432 1.51 (0.53–4.28)
Performance status (ECOG score)	1 and 2	0.4828 0.65 (0.20–2.15)	0.4706 0.65 (0.20–2.08)	0.1017 2.40 (0.84–6.83)
Smoking status	Smoker	0.75610.85 (0.32–2.29)	0.87051.08 (0.41–2.88)	0.28981.65 (0.66–4.13)
Packyears [years]	≥45	0.4635 1.21 (0.72–2.04)	0.1773 1.42 (0.86–2.35)	0.9711 0.99 (0.60–1.63)
1st line CHTH (I)	Cis + Pem	0.2384 0.72 (0.42–1.24)	0.1095 0.64 (0.37–1.10)	0.4136 0.80 (0.47–1.36)
	PN	0.2657 0.63 (0.28–1.42)	0.0953 0.50 (0.22–1.12)	0.0652 0.61 (0.36–1.03)
	PG	0.2938 1.55 (0.69–3.51)	0.118 1.94 (0.86–4.39)	0.0915 1.90 (0.91–3.99)
Number of chemotherapy cycles	>3	<0.0001 * 0.33 (0.20–0.56)	0.001 * 0.36 (0.22–0.60)	0.0056 * 0.49 (0.30–0.81)
Distant metastases	Yes	0.0545 3.41 (0.98–11.83)	0.0363 * 3.76 (1.09–12.89)	0.3057 1.74 (0.61–4.96)
Distant metastases (CNS)	Yes	<0.0001 * 6.45 (2.86–14.57)	0.0003 * 4.22 (1.93–9.21)	<0.0001 * 6.18 (2.64–14.45)
Distant metastases (Bones)	Yes	0.0005 * 2.91 (1.59–5.31)	0.0006 * 2.92 (1.59–5.37)	0.0042 * 2.33 (1.31–4.13)
Distant metastases (second lung)	Yes	0.0046 * 2.97 (1.40–6.29)	0.0035 * 3.16 (1.15–6.81)	0.0013 * 3.36 (1.61–7.00)
Distant metastases (andrenal glands)	Yes	<0.0001 * 12.15 (3.95–37.38)	<0.0001 * 17.14 (4.95–59.37)	0.0085 * 3.40 (1.37–8.43)
Distant metastases (liver)	Yes	0.0003 * 8.03 (2.58–24.99)	0.0001 * 10.88 (3.33–35.56)	0.2448 2.05 (0.61–6.83)
Distant metastases (distant lymph nodes)	Yes	0.0035 * 3.13 (1.46–6.71)	0.0040 * 3.09 (1.44–6.63)	0.0040 * 3.19 (1.45–7.02)
BMI [kg/m^2^]	<24.91	0.9437 1.02 (0.61–1.70)	0.7581 1.08 (0.65–1.82)	0.0861 1.55 (0.94–2.55)
Body loss [%]	≥0.84	0.9617 1.02 (0.54–1.92)	0.7046 1.13 (0.61–2.09)	0.2065 1.40 (0.83–2.36)
Time from diagnosis to treatment [days]	≥17	0.2204 0.73 (0.44–1.21)	0.9892 1.00 (0.61–1.66)	0.5575 1.16 (0.71–1.89)
Occupational exposure	Yes	0.1792 1.77 (0.77–4.04)	0.0828 2.09 (0.91–4.80)	0.7594 1.13 (0.51–2.52)
Family history of any cancer	Yes	0.3321 1.29 (0.77–2.14)	0.7056 1.10 (0.67–1.19)	0.2663 0.75 (0.46–1.24)
Family history of lung cancer	Yes	0.4613 0.71 (0.29–1.75)	0.7610 0.87 (0.36–2.11)	0.8907 1.06 (0.46–2.43)
Histopathology	SCC	0.0138 * 2.85 (1.24–6.54)	0.0077 * 3.09 (1.35–7.07)	0.0406 * 2.10 (1.04–4.28)
*BIRC5* (rs8073069)	CC	0.0181 * 0.28 (0.10–0.80)	0.0125 * 0.28 (0.10–0.76)	0.1629 0.50 (0.19–1.32)
	GG	0.0136 * 1.92 (1.15–3.24)	0.0332 * 1.74 (1.05–2.89)	<0.0001 * 3.63 (2.05–6.43)
	CG	0.4169 0.81 (0.49–1.34)	0.7987 0.94 (0.57–1.54)	0.1629 2.00 (0.76–5.31)

Abbreviations: BMI—body mass index; SCC—squamous cell carcinoma; *—statistically significant. Results of multivariate analysis were adjusted for age (PFS, TTP), number of cycles, histopathology and *BIRC5* SNP (PFS, TTP, OS) as well as distant metastases (TTP). In some cases (*n* = 12 for PFS and TTP; *n* = 9 for OS), reliable determination of the survival time was not possible to estimate (too short follow-up—discontinuation of treatment prior to assessment time because of poor tolerance, failure to appear at the next appointment, or any contact with a patient was lost).

## Data Availability

The data used to support the findings of this study are available from the corresponding authors upon request.

## Supplementary Materials

The following supporting information can be downloaded at: https://www.mdpi.com/article/10.3390/cells11060956/s1, Table S1. Distribution of survivin (rs8073069) genotypes according to demographic and clinical factors.
