# Supplementary material for: Polymorphism of Baculoviral Inhibitor of Apoptosis Repeat-Containing 5 (BIRC5) Can Be Associated with Clinical Outcome of Non-Small Cell Lung Cancer
Source: Cells. 2022 Mar 10;11(6):956. doi: 10.3390/cells11060956 (PMC8946487; doi:10.3390/cells11060956)
Supplement: Supplementary file 1 [file cells-11-00956-s001.zip › cells-1607319-supplementary.pdf]

Table S1. Distribution of *BIRC5* (rs8073069) genotypes according to demographic and clinical factors.

**Variable**		***Survivin* (rs8073069)**			
		**CC**	**CG**	**GG**	***p***
		***n* = 11 (10.6%)**	***n* = 48 (46.2%)**	***n* = 45 (43.3%)**	
**Sex**	Male	7 (9%)	33 (42.3%)	38 (48.7%)	0.1424
	Female	4 (15.4%)	15 (57.7%)	7 (26.9%)	
**Age [years]**	≥ 66.5	3 (5.9%)	24 (47.1%)	24 (47.1%)	0.2959
	< 66.5	8 (15.1%)	24 (45.3%)	21 (39.6%)	
**Disease stage**	III	4 (10.5%)	16 (42.1%)	18 (47.4%)	0.8004
	IV	7 (10.6%)	32 (48.5%)	27 (40.9%)	
**Distant metastases**	No	4 (8.7%)	19 (47.8%)	22 (43.5%)	0.5891
	Yes	7 (12.1%)	29 (48.3%)	23 (39.6%)	
**Histopathology**	AC	4 (9.3%)	21 (48.8%)	18 (41.9%)	0.1213
	SCC	5 (9.3%)	22 (40.7%)	27 (50%)	
	NOS	2 (28.6%)	5 (71.4%)	-	
**Performance status (ECOG score)**	0	3 (30%)	9 (50%)	6 (20%)	0.5321
	1	4 (6%)	26 (44.8%)	29 (49.2%)	
	2	4 (14.8%)	13 (48.2%)	10 (37%)	
**Smoking status**	Smoker	9 (9.3%)	46 (47.4%)	42 (43.3%)	0.2465
	Non-smoker	2 (28.6%)	2 (28.6%)	3 (42.9%)	
**Body loss [%]**	≥ 10%	4 (11.4%)	13 (37.1%)	18 (51.4%)	0.4115
	< 10%	7 (10.1%)	35 (50.7%)	27 (39.1%)	

**Occupational exposure**	No	10 (11.1%)	43 (47.8%)	37 (41.1.%)	0.5268
	Yes	1 (7.1%)	5 (35.7%)	8 (57.1%)	
**Family history of any cancer**	No	2 (4.2%)	23 (47.9%)	23 (47.9%)	0.1375
	Yes	9 (16.1%)	25 (44.6%)	22 (39.3%)	
**Family history of lung cancer**	No	6 (7.8%)	38 (49.4%)	33 (42.9%)	0.2414
	Yes	5 (18.5%)	10 (37%)	12 (44.4%)	

Abreviations: AC, adenocarcinoma; NOS, not otherwise specified; SCC, squamous cell carcinoma; ECOG, Eastern Cooperative Oncology Group.
